# IMMUNE AGING AT SINGLE-CELL RESOLUTION

**DOI:** 10.1093/geroni/igad104.1426

**Published:** 2023-12-21

**Authors:** Maxim Artyomov

**Affiliations:** Washington University in St. Louis, St. Louis, Missouri, United States

## Abstract

I will discuss how single-cell resolution approaches reveal aspects of immune aging in tissues and in circulation. We will discuss comparison of immune aging in mouse tissues, accumulation of GZMK+ CD8 T cells and their human counterparts that accumulate in blood. We will discuss analysis of scRNA-seq profiling of PBMC along the healthy aging (317 samples from 25 to 85yo) and emerging subpopulation and antigen receptor changes during aging.

